# Kimura’s Disease: A Case Report

**DOI:** 10.7759/cureus.90446

**Published:** 2025-08-18

**Authors:** Karolina Akinosoglou, Stamatia Tsoupra, Aggelos Rigopoulos, Georgios Schinas, Eleni Polyzou, Ioanna Akrida, Vasiliki Labropoulou, Argiris Symeonidis, Georgia Kaiafa, Eleni Kourea

**Affiliations:** 1 Department of Internal Medicine and Infectious Diseases, University General Hospital of Patras, University of Patras, Patras, GRC; 2 Department of Internal Medicine, University General Hospital of Patras, University of Patras, Patras, GRC; 3 Department of Medicine, University of Patras, Patras, GRC; 4 Department of Surgery, University General Hospital of Patras, University of Patras, Patras, GRC; 5 Department of Hematology, Department of Medicine, University General Hospital of Patras, University of Patras, Patras, GRC; 6 Department of Hematology, Department of Internal Medicine, University General Hospital of Patras, University of Patras, Patras, GRC; 7 Department of Medicine, First Propaedeutic Department of Internal Medicine, AHEPA Thessaloniki University General Hospital, Aristotle University of Thessaloniki, Thessaloniki, GRC; 8 Department of Pathology, Department of Medicine, University General Hospital of Patras, University of Patras, Patras, GRC

**Keywords:** elevated ige, eosinophilia, head and neck lesions, kimura’s disease, lymphadenopathy

## Abstract

Kimura's disease (KD) is a rare, chronic, autoinflammatory condition of unknown etiology, typically involving lymphoid and/or extranodal tissues of the head and neck area, usually presenting with peripheral blood eosinophilia and elevated serum immunoglobulin E (IgE) levels. The clinical features of KD are often variable and nonspecific, overlapping with other hematologic conditions, leading to diagnostic challenges. We report a case of a 36-year-old female with no prior medical history who presented with bilateral cervical lymphadenopathy, nodular lesions in the scalp, and elevated IgE levels, diagnosed with KD. We present a scoping review of the literature focusing on mass-like presentations of the head and neck region, recording the demographics, the clinical manifestation, the histopathology, and imaging findings, as well as the treatment and the outcomes. This case report and literature review highlight the necessity of clinical awareness of this rare condition and emphasize the need for interdisciplinary collaboration in the diagnostic process.

## Introduction

Kimura's disease (KD) is a rare, chronic, autoinflammatory condition of unknown etiology, characterized by lymphoid and/or extranodal tissue involvement, peripheral blood eosinophilia, and elevated serum immunoglobulin E (IgE) levels [1]. Although once believed to be the same condition, angiolymphoid hyperplasia with eosinophilia (ALHE) and KD are now recognized as separate entities, differing both clinically and histologically [2].

KD predominantly affects young Asian males, with a male-to-female ratio of 3:1 [3]. The exact pathogenesis remains unclear, although an autoimmune etiology or a hypersensitivity reaction has been proposed [4]. Due to its rarity and nonspecific presentation, the diagnosis of KD can be challenging [3].

This report presents a case of KD in a 36-year-old female with no prior medical history who presented with cervical and supraclavicular lymphadenopathy and nodular lesions in the scalp. It also reviews the literature on mass-like presentations of the head and neck region, highlighting the importance of considering KD in the differential diagnosis after excluding lymphoproliferative disorders, metastatic malignancy, and infectious causes.

## Case presentation

**Figure 1:**
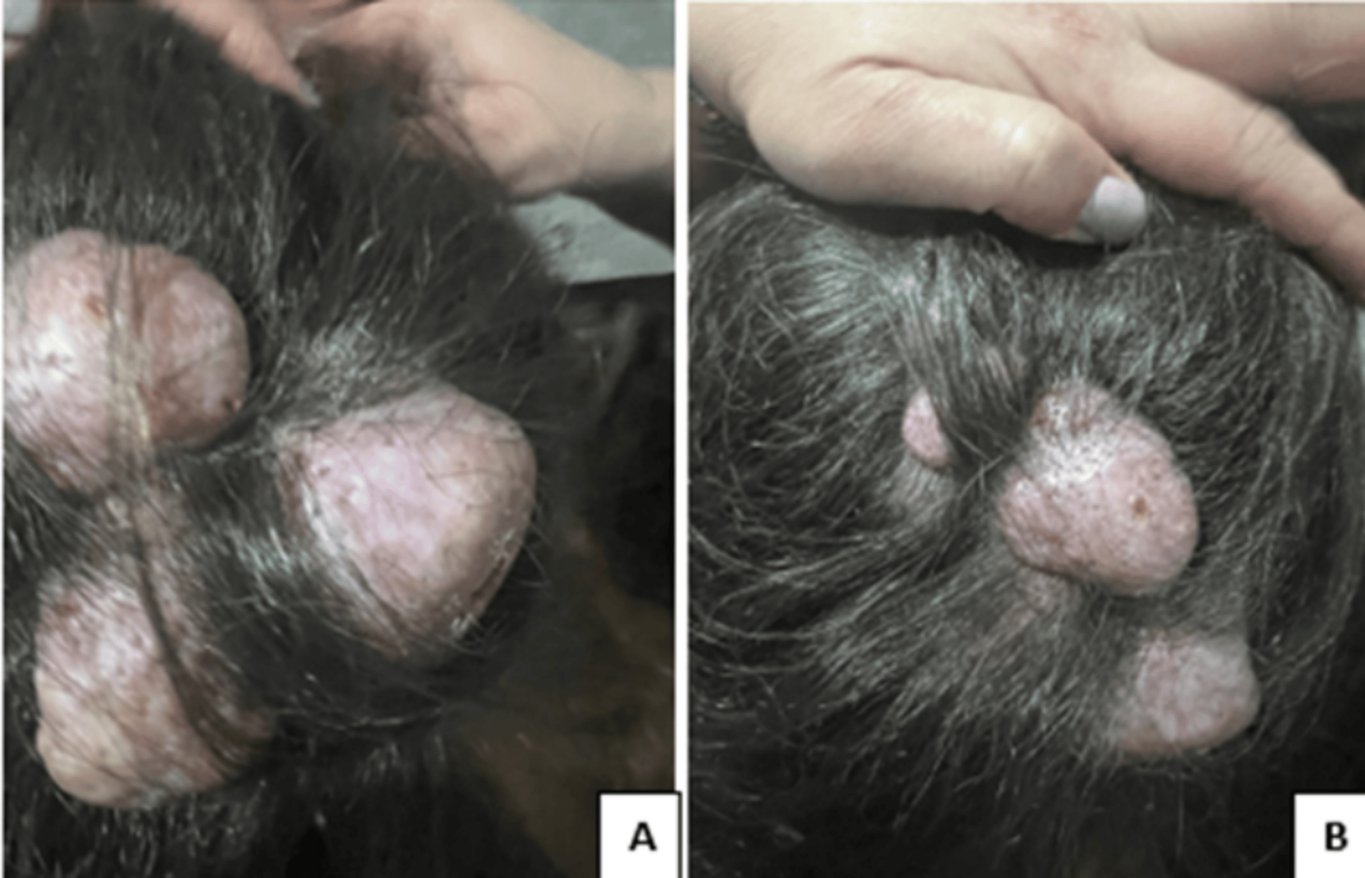
Multiple lesions of the lower parietal (A) and occipital (B) regions of the scalp (4-5 cm) Photo release: The patient has given consent for ηερ photograph to be used in this case report for the purpose of case presentation as per the respective ethics research committee and respective institutional review board approval (05/08/2024).

A 36-year-old female with no prior medical history presented with bilateral, painless, hard cervical, and supraclavicular lymphadenopathy (4-5 cm) occurring during the last year. The patient had an unremarkable social and family history and was in good general condition. Physical examination revealed multiple nodular, pruritic, subcutaneous lesions (4-5 cm) in the lower parietal and occipital regions of the scalp, which had been present for 20 years (Figure 1). No other symptoms or findings were found during the examination.

**Figure 2:**
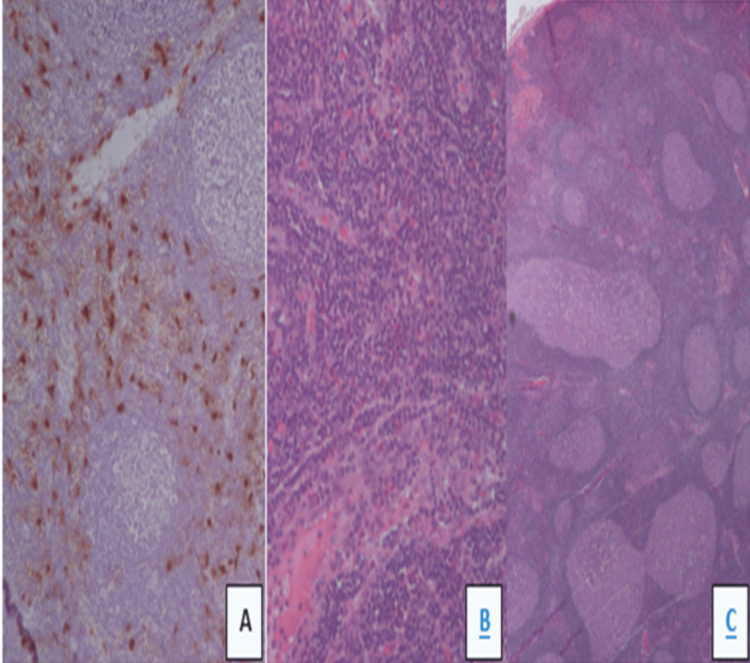
Histopathological features of supraclavicular lymph node (A). The lymph node biopsy shows prominent follicular hyperplasia with irregularly shaped reactive germinal centers. (B) In the interfollicular areas, a vascular proliferation with increased numbers of eosinophils are noted. (C) Immunohistochemical stain for tryptase highlights the increased numbers of mast cells in the interfollicular areas

The diagnostic workup revealed marginal eosinophilia and markedly elevated IgE levels in the absence of an oligoclonal band. Testing for infectious causes, including HIV, *Toxoplasma*, and *Mycobacterium *sp., was negative. Cancer, inflammatory biomarkers, and angiotensin-converting enzyme (ACE) levels were normal. Positron emission tomography-computed tomography (PET-CT) confirmed local lymphadenopathy in the absence of other foci. The lymph node biopsy showed prominent follicular hyperplasia with irregularly shaped reactive germinal centers (Figure 2A). In the interfollicular areas, a vascular proliferation with increased numbers of eosinophils was noted (Figure 2B). Immunohistochemical stain for tryptase highlighted the increased numbers of mast cells in the interfollicular areas (Figure 2C). The combination of clinical findings, laboratory results, and histopathological features established the diagnosis of KD.

Management

The patient was treated with systemic corticosteroids, which significantly reduced the size of the lymphadenopathy and improved pruritus. The patient remains under close follow-up to monitor disease progression and potential complications, including nephrotic syndrome.

Case discussion and literature review

This case highlights the importance of considering KD in the differential diagnosis of mass-like presentations in the scalp and head region. The rare presentation and feature overlap with various hematologic conditions, often leading to misdiagnosis or delayed diagnosis. Thus, we performed a review in order to provide insights into the clinical presentations, diagnostic challenges, and management strategies for KD.

Methods

A comprehensive literature review was conducted to identify studies reporting on KD, with a focus on mass-like presentations of the head and neck region, excluding ophthalmologic manifestations. The following databases were searched from inception until March 2025: PubMed, Embase, Scopus, and Web of Science. The search strategy involved the use of controlled vocabulary (MeSH terms) and free-text terms for the concept of KD. The search query basis included the following terms: ("Kimura disease" OR "Kimura's" OR "Kimura") AND ("head and neck" OR "mass" OR "scalp" OR "skin"). Additional relevant articles were identified by manually searching the reference lists of the included studies.

**Figure 3:**
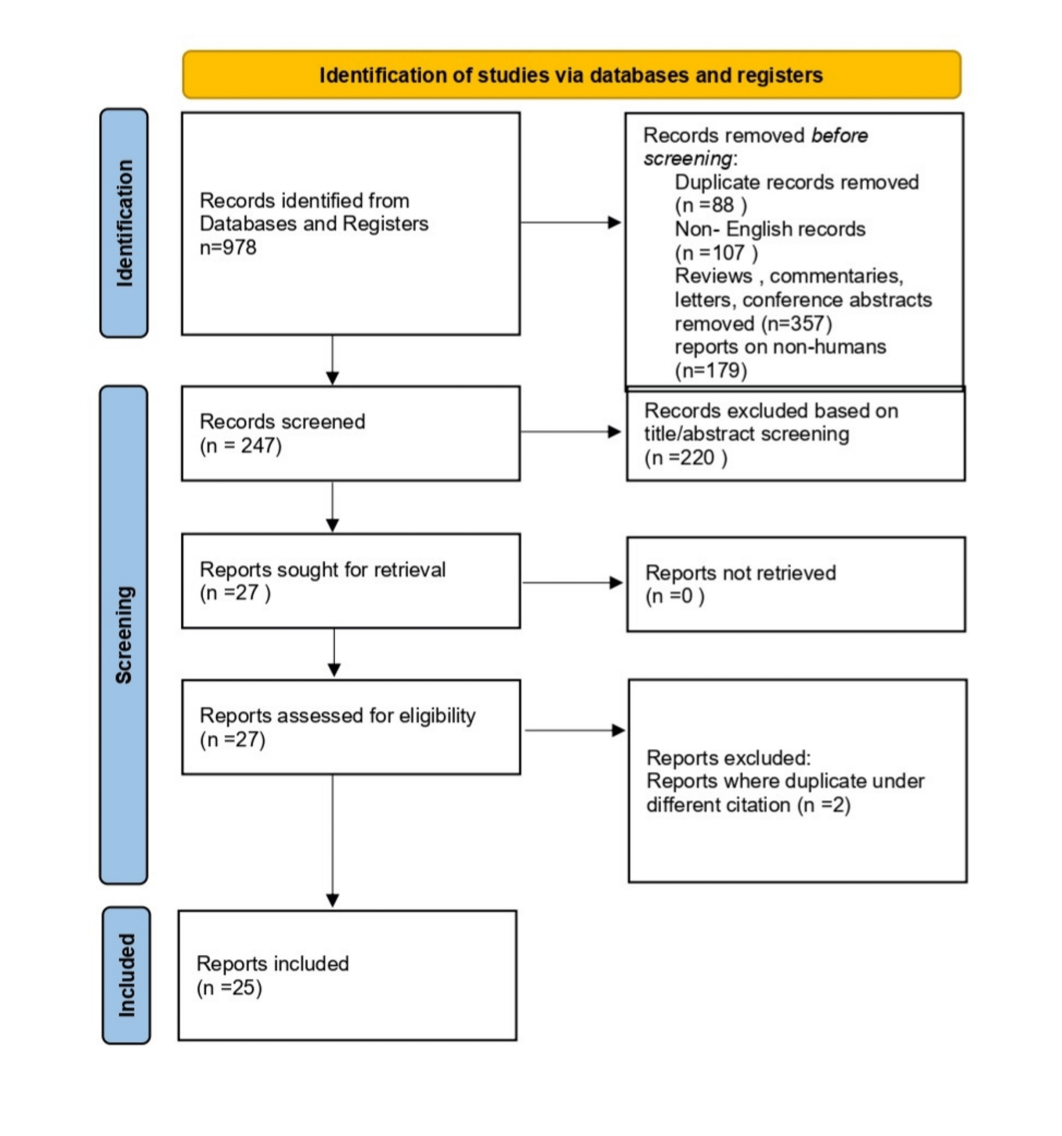
Flowchart of the study selection

Eligibility criteria for study selection were as follows: (1) case reports, or case series reporting on patients diagnosed with KD presenting with mass-like lesions in the head and neck region; (2) articles published in English; and (3) articles providing sufficient data on patient demographics, clinical presentation, diagnostic workup, and management strategies. Reviews, commentaries, letters, and conference abstracts were excluded. Two reviewers independently assessed the eligibility of the identified studies, and any disagreement was resolved through discussion and consensus (Figure 3).

Data extraction was performed independently by two reviewers using a standardized data extraction form. The extracted information included study design, publication year, patient demographics (age, gender), clinical presentation, diagnostic workup (laboratory findings, imaging, histopathology), and management strategies. The extracted data were synthesized and presented in a narrative format. A summary table (Table 1) was constructed to provide an overview of the literature on KD in the context of mass-like presentations in the head and neck region.

**Table 1:** Cases of Kimura's disease

Reference/Case	N	Sex	Age	Clinical Presentation	Imaging Findings	Pathology Findings	Treatment Description	Outcome Description
Kumar et al. [3] (2018), Case 2	1	M	29	Progressive unilateral cervical, supraclavicular, mediastinal, hilar, axillary lymphadenopathy, mild pain	MRI: cervicomediastinal mass	Lymphoid hyperplasia, eosinophils, fibrosis, atypia	Glucocorticoids, chemotherapy	Clinical improvement
Gupta et al. [5] (2019), Case 1	1	F	36	2-Year swelling, right pinna, painless, mobile, non-tender	CT: intra-parotid and jugular nodes	Lymphoid hyperplasia, eosinophils	Surgery	Free from disease for 1 year
Gupta et al. [5] (2019), Case 2	1	M	30	1-Year swelling postauricular, painless, mobile	US: hypoechoic lesion	Eosinophils, microabscess, fibrosis	Surgery	Free from disease at follow-up
Ye et al. [6] (2017)	46	40 M, 6 F	n/s	Parotid, submandibular, submental, cervical swelling, some with pigmentation, pruritus, nephrotic syndrome	US: hypoechoic masses or nodes	Eosinophilia	Surgery, radiotherapy	Recurrences: surgery 12, surgery+Radiotherapy 2, Radiotherapy 2
Punia et al. [7] (2013)	8	6 M, 2 F	n/s	Neck, preauricular, eyebrow, mandible, parotid, postauricular swelling, variable symptoms	US: subcutaneous lesion	Lymphoid hyperplasia, eosinophils, microabscess, fibrosis	Not stated	Not stated
Zhang et al. [8] (2018)	12	11 M, 1 F	49.3	Slow-growing nodules, parotid, neck, cheek, submaxillary, postauricular, some pruritus	CT: lymphadenectasis, parotid lesion	Lymphoid hyperplasia, eosinophils, fibrosis	Surgery, radiotherapy	Not stated
Glibbery et al. [9] (2019)	1	F	41	Intermittent left parotid swelling, urticarial/eczematous lesions, trunk and limbs	PET: increased uptake, CT: soft tissue	Follicular hyperplasia	Antibiotics, cetirizine, parotidectomy	Not stated
Eh Dam et al. [10] (2020)	1	M	16	Painless infra-auricular and parotid swelling, multiple lymph nodes	CT: heterogeneous mass	Lymphadenitis, atypia	Prednisolone, parotidectomy	Not stated
Woo et al. [11] (2017)	1	M	33	Bilateral parotid swelling for 10 years	CT: non-homogeneous mass	Lymphoid hyperplasia, eosinophils	Excision of masses	Not stated
Sousa et al. [12] (2020)	1	M	19	Left facial swelling for 1.5 years	MRI: lesion in buccal space	Lymphoid hyperplasia, fibrosis	Excision of mass	Not stated
Faras et al. [13] (2014)	1	M	33	Left parotid swelling for 12 years	CT: parotid enlargement, MRI: enlarged parotids	Inflammation, germinal centers, fibrosis	Steroids, parotidectomy	Relapse after steroids
Malhorta et al. [14] (2017)	1	M	40	Swelling behind the right ear for 6 years	Not stated	Lymphoid hyperplasia	Prednisone, tapered	Reduced after 8 weeks
Sato et al. [15] (2021)	1	M	52	Left buccal swelling for 2 years	CT: mass with edema, MRI: solid mass	Lymphoid hyperplasia, eosinophils	Prednisolone, tapered	Not stated
Sneha et al. [16] (2015)	1	M	8	Swelling behind the ears for 2 years, itching for 3 months, lymphadenopathy	Not stated	Vascularisation, giant cells	Observation, follow-up	Stable
Bishop et al. [17] (2022)	1	M	39	Right facial mass with pain for 2 years	CT, MRI: enhancing mass	Lymphoid, eosinophils, fibrosis	Surgery, corticosteroids	Not stated
Alanazi et al. [18] (2022)	1	M	40	Painless, firm right preauricular mass for 2 years, sickle cell disease	CT: heterogeneous parotid, MRI: mass	Lymphoid hyperplasia, eosinophils, fibrosis	Parotidectomy	Not stated
Lee et al. [19] (2001)	1	M	16	Painless right buccal mass for 2 years, nephrotic syndrome	CT: nodular enhancing lesion	Follicular hyperplasia, eosinophils	Surgical resection of mass	Not stated
Gupta et al. [20] (2016)	1	F	27	Small nodular right postauricular swelling for 6 months	Not stated	Follicular hyperplasia, eosinophils, giant cells	Surgical resection of mass	Not stated
AlGhambi et al. [21] (2016)	1	M	11	Right facial mass for 5 years, non-painful	CT: soft tissue mass, MRI: high T2 signal	Germinal centers, fibrosis	Loratadine, prednisolone	Mass reappeared after stopping steroids
Sharma et al. [22] (2017)	1	M	23	Two painless swellings (right cheek, left temple), 6 years	MRI: mass, enlarged cervical nodes	Lymphocytes, eosinophils	Parotidectomy, prednisolone	Not stated
Alsmoudi et al. [23](2024)	1	M	23	Left and right periauricular swelling, pain for 7 months, itching, fever, fatigue, and weight loss	Not stated	Lymphocytes, no abnormal cells	Cyclosporine, antihistamines, steroids	Not stated
Lee et al. [24] (2005)	1	M	16	Left postauricular mass, bilateral lacrimal gland swelling for 3 years	CT: rectus muscles, lacrimal gland enlarged	Lymphoid hyperplasia, eosinophils	Steroid treatment	Not stated
Prayuenyong et al. [25] (2025)	1	M	22	Left parotid swelling for 3 years	US, CT: enlarged intraparotid and neck nodes	Lymphoid hyperplasia	Parotidectomy	Not stated
Majdi et al. [26] (2025)	1	M	31	Firm, mobile parotid mass for 6 years	MRI: nodular lesion, hypointense T1, hyperintense T2	Lymphocytes, eosinophils	Exofacial parotidectomy	No recurrence at 3 years

Results

Among 87 patients with head and neck involvement, the majority were male (86.2%), with a mean age of 35.7 years. The most common imaging findings were parotid or neck masses on CT (14.9%), followed by MRI-detected parotid or facial masses (9.2%) and hypoechoic lesions on ultrasound (8.0%). Histologically, lymphoid hyperplasia with eosinophilic infiltration was the predominant biopsy finding, present in 31.0% of cases. Other features included follicular or paracortical hyperplasia (8.0%), sclerotic stroma and fibrosis (8.0%), and eosinophilic microabscesses (3.4%). Nearly half of the patients (47.1%) underwent surgery, making it the most frequent treatment, followed by steroid therapy (17.2%) and combined surgery with radiotherapy (5.7%). Outcomes were generally favorable, with most patients achieving disease control or remission, though relapses were noted, especially after discontinuation of steroids.

## Discussion

Epidemiology

KD is a rare chronic autoinflammatory condition primarily affecting males of Asian descent but also occurring in other ethnicities [1,27]. In China and Japan, KD has been referred to as "eosinophilic hyperplastic lymphogranuloma" or as "atypical granulation linked to hyperplastic changes in lymphoid tissue". The exact prevalence and incidence of KD remain unclear. It primarily affects young males of East Asian descent, with the highest occurrence typically seen in the second and third decades of life [1]. While endemic to Asia, cases have been reported in Europe and America [27], while cases in young children have also been recorded [28,29]. Of note, the male:female ratio seems to decrease with age [28]. Herein, the reviewed studies encompassed patients with varying age and sex profiles. The ages of the patients ranged from 8 to 62 years old [28,30]. While the majority of the cases involved male patients, there was also a notable number of female patients reported in these studies [31,32]. The ethnicity of patients varied [1], with cases reported from different continents, including Asia [33,34], Europe [35,36], Africa [37,38], and North and South America [3]. This highlights that KD can affect individuals from various age groups, races, and both sexes, although it is predominantly seen in males with a slow disease progression over months to years.

Pathophysiology

The pathogenesis of KD is unknown. Trauma, infection, an IgE-mediated hypersensitivity reaction, or an autoimmune process have been postulated as possible causes. The pathophysiology involves an aberrant immune reaction to an unknown stimulus, with IgE-positive mast cells and IgE reticular networks in germinal centers [39]. KD shares features with cutaneous IgG4-related disease, suggesting a potential pathophysiological link [40]. Its pathogenesis seems to involve complex immune interactions, with studies revealing overactivation of the Erk/MAPK signaling pathway in eosinophils and loss of S100P in CD24+ myeloid cells [41]. Tissue infiltration by CD4+ GATA3+ T cells and IL-4+ IgE+ c-kit+ mast cells suggests an IgE-mediated allergic response [42]. While its etiology remains unknown, clonal T cell populations have been identified in some cases, potentially contributing to disease pathogenesis [43].

Clinical manifestations

With regards to clinical manifestations, cases of KD primarily present as painless, mass-like findings in the head and neck region, frequently with the involvement of the peripheral lymph nodes and the parotid gland [3]. The patients' specific affected sites, sizes, numbers, and general clinical appearances of the nodular masses vary. The extent of involvement, as seen in imaging studies, remains diverse across patients. KD can also affect extracutaneous sites, such as the parotid gland, orbit, oral cavity, and nasal sinuses [44]. Uncommon skin manifestations may include generalized itching, eczema, and prurigo nodularis, while systemic symptoms are typically not present [45]. The incidence of pruritus in KD increases with age, occurring in 3.8% of patients under 20 years, 15.5% of those aged 20-39 years, and 21.7% of those aged 40 and above [28]. This age-related rise in pruritus may be partially linked to age-associated changes in humoral and cellular immunity, including eosinophilia and elevated IgE levels [46]. Renal involvement has been observed in about 20% of patients with KD, including conditions such as minimal change disease, mesangial proliferative glomerulonephritis, and membranous nephropathy. Additionally, approximately 12%-16% of patients go on to develop nephrotic syndrome [47].

In our report, some patients had unilateral involvement, while others had bilateral or multiple-site involvement or lymphadenopathy [1]. There was also a spectrum of associated symptoms among the patients, including pain, itching, and systemic manifestations. The differential diagnoses for these presentations include lymphoproliferative disorders, malignancy, infectious causes, granulomatous diseases, and autoimmune conditions [5]. Diagnostic challenges can include differentiating KD from other causes of head and neck masses, such as lymphoma, angiolymphoid hyperplasia with eosinophilia (ALHE), and benign/reactive lymphadenopathy [3]. In our case, the absence of fever, weight loss, tender or generalized lymphadenopathy, exposure to known risk factors, or musculoskeletal complaints made the above conditions less likely.

Diagnosis

The diagnosis of KD is based on clinical presentation, laboratory results, and histopathological findings [7]. In several cases, the laboratory examinations showed peripheral eosinophilia and elevated IgE serum levels. In some cases, proteinuria may precede the appearance of skin lesions [48].

In addition, there is a critical role of imaging studies such as ultrasound (US), CT, and MRI in the diagnostic process due to the diversity of KD’s diagnostic findings. CT and MRI typically reveal subcutaneous lesions, infiltrative parotid masses, and cervical lymphadenopathy [49,50]. Diffusion-weighted imaging (DWI) shows higher apparent diffusion coefficient (ADC) values in subcutaneous and parotid lesions compared to normal tissue, while reactive lymph nodes exhibit lower ADC values [51,52]. Dynamic contrast-enhanced MRI demonstrates gradual upward enhancement in subcutaneous lesions, distinguishing them from malignancies [50]. MR spectroscopy reveals slightly elevated choline/creatine ratios in affected areas [51]. The integration of multiple imaging techniques, particularly DWI and dynamic contrast-enhanced MRI, enhances diagnostic accuracy for this rare condition and reinforces the utility of combining DWI and dynamic contrast-enhanced MRI to distinguish KD-related lesions from malignancies and benign reactive nodes, supporting their integration in routine diagnostic workflows.

In combination with the clinical features and laboratory findings, the diagnosis is established by the histopathological examination [1,3]. The pathology findings from the presented studies consistently demonstrate several key features characteristic of KD. Histopathological examinations frequently revealed follicular hyperplasia, germinal centers, and eosinophilic infiltration. However, histopathological features differed in the extent of eosinophilic infiltration, fibrosis, and vascular proliferation. Many cases presented with enlarged lymph nodes with reactive germinal centers and lymphoid follicles, thus indicating an immune response and lymphoid tissue proliferation [7]. A prominent eosinophilic infiltrate was found in the affected tissues across multiple studies, with some cases of eosinophilic microabscesses and deposits within germinal centers [1]. Several studies reported increased vascularization within germinal centers, as well as the presence of hyalinized vessels, indicating an angiogenic process in the affected tissue [2]. Dense and interstitial fibrosis was noted, suggesting tissue remodeling and scarring in the affected areas. In a few studies, CD30(+) atypical cells and Reed-Sternberg cells were detected, which may indicate a possible overlap with other hematologic conditions, such as Hodgkin's lymphoma [53]. The similarities and differences among these case reports highlight the need for a high index of clinical suspicion and a comprehensive diagnostic approach to diagnose and manage this rare entity accurately.

Management

There is no standardized treatment protocol for KD. For localized lesions, surgical excision is typically the first-line therapy. In cases involving large lesions that are challenging to remove completely and carry a high risk of recurrence, surgical excision followed by low-dose postoperative radiation therapy has been suggested as a potential treatment strategy [54]. Treatment options include immunosuppressive therapy with systemic corticosteroids or immunomodulatory agents, such as cyclosporine, mycophenolate mofetil [20,55,56], or mycophenolic acid [57], and a combination of leflunomide with methylprednisolone [58] has been used in select cases, particularly in patients with multifocal disease or concurrent nephrotic syndrome. Meanwhile, long-term corticosteroid treatment was reported in some cases [59]. Targeted biologic therapies have also shown promise in isolated cases. Interleukin-5 (IL-5) blockade with mepolizumab demonstrated effectiveness and good tolerability in several case reports [60], while IL-4 and IL-13 blockade using subcutaneous dupilumab has also been reported as beneficial in treating KD. Surgical excision, radiotherapy, or a combination of these modalities is also reported [6]. However, none of these treatments have been evaluated in randomized controlled trials, and current evidence is limited to small case series and individual case reports.

Prognosis and follow-up

Recurrence of KD is common, regardless of the treatment approach. In a review of 25 cases, recurrence rates ranged from 20% to 100% following both surgical and medical therapies [40]. A pooled analysis of 22 small case series involving 570 patients found that those treated with surgical excision alone had a fivefold higher risk of recurrence compared to those who received surgery followed by radiation therapy [54]. Prognostic factors suggesting disease recurrence include eosinophils >50%, serum IgE >10,000 IU/mL, and multifocal lesions outside salivary glands [61]. Although the condition rarely resolves spontaneously, the overall prognosis is good, with no cases of malignant transformation reported [62,63]. Prognosis in patients with renal involvement varies based on the type and severity of nephropathy. Notably, one case reported remission of membranous nephropathy following surgical removal of a left axillary mass in a patient with KD [64].

In our report, the choice of treatment and the subsequent outcome varied. Some cases reported successful disease control with surgery alone, while others required a combination therapy with corticosteroids or radiotherapy [65,66]. Disease recurrence or relapse was observed in a few cases. This fact underlines the importance of long-term monitoring of patients with KD and highlights the collaboration among medical specialties in the diagnosis and management of rare diseases, especially in patients with atypical presentations.

## Conclusions

The literature review focuses on the diagnostic challenges and management strategies of KD, particularly in patients presenting with mass-like lesions in the head and neck region. We provided a comprehensive and clinically relevant resource for healthcare professionals who may encounter this rare condition by focusing on KD's diagnostic challenges and management strategies. The similarities and differences among these case reports highlight the need for a high index of clinical suspicion and a comprehensive diagnostic approach to accurately diagnose and manage this rare entity. This case report and literature review underscore the need for interdisciplinary collaboration in the diagnostic process to ensure accurate diagnosis and appropriate management.
